# No consistent startle modulation by reward

**DOI:** 10.1038/s41598-021-82902-0

**Published:** 2021-02-23

**Authors:** Iris Schutte, Johanna M. P. Baas, Ivo Heitland, J. Leon Kenemans

**Affiliations:** 1 Department of Experimental Psychology, Helmholtz Institute, Utrecht University, Utrecht, The Netherlands; 2 Klinik Für Psychiatrie, Sozialpsychiatrie Und Psychotherapie, Medizinische Hochschule Hannover, Hannover, Germany

**Keywords:** Motivation, Human behaviour

## Abstract

Previous studies have not clearly demonstrated whether motivational tendencies during reward feedback are mainly characterized by appetitive responses to a gain or mainly by aversive consequences of reward omission. In the current study this issue was addressed employing a passive head or tails game and using the startle reflex as an index of the appetitive-aversive continuum. A second aim of the current study was to use startle-reflex modulation as a means to compare the subjective value of monetary rewards of varying magnitude. Startle responses after receiving feedback that a potential reward was won or not won were compared with a baseline condition without a potential gain. Furthermore, startle responses during anticipation of no versus potential gain were compared. Consistent with previous studies, startle-reflex magnitudes were significantly potentiated when participants anticipated a reward compared to no reward, which may reflect anticipatory arousal. Specifically for the largest reward (20-cents) startle magnitudes were potentiated when a reward was at stake but not won, compared to a neutral baseline without potential gain. In contrast, startle was not inhibited relative to baseline when a reward was won. This suggests that startle modulation during feedback is better characterized in terms of potentiation when missing out on reward rather than in terms of inhibition as a result of winning. However, neither of these effects were replicated in a more targeted second experiment. The discrepancy between these experiments may be due to differences in motivation to obtain rewards or differences in task engagement. From these experiments it may be concluded that the nature of the processing of reward feedback and reward cues is very sensitive to experimental parameters and settings. These studies show how apparently modest changes in these parameters and settings may lead to quite different modulations of appetitive/aversive motivation. A future experiment may shed more light on the question whether startle-reflex modulation after feedback is indeed mainly characterized by the aversive consequences of reward omission for relatively large rewards.

## Introduction

Appetitive and aversive motivations are fundamental for human functioning as they promote automatic behavioral tendencies and adaptive responses in the context of reward and punishment^1,2^. A large body of research has shown that the startle eye blink reflex captures motivational direction (i.e., appetitive or aversive oriented motivation) within the context of emotional picture-viewing^3^. More specifically, the startle eyeblink reflex is typically potentiated in the context of an aversive stimulus and inhibited in the context of an appetitive stimulus, e.g.^4–6^. These effects may be explained by a relative match or a mismatch between the subject’s motivational state induced by the emotional context and the defensive nature of the startle reflex^7^. In line with this theory, it has been shown that startle-reflex modulation is associated with self-reported sensitivity to reward. Startle magnitudes during positive picture-viewing were more strongly reduced in high- compared to low reward sensitive individuals^8^. Furthermore, it has been found that manipulation of approach motivation by leaning forward causally influences startle modulation during appetitive events^9^. In the current study we used startle-reflex modulation as a means to assess appetitive and aversive motivational drives during the obtainment and loss of monetary rewards of varying magnitude.

A number of prior studies investigated startle-reflex modulation after obtainment and loss of monetary^10^ or food rewards^11^. Consistent with the motivational priming theory described above^7^, these studies observed relative startle inhibition after obtainment of reward compared to loss of reward or punishment. These reports claim that emotional modulation of the startle reflex (i.e., a difference in startle magnitude between the reward obtainment and loss condition) is explained by obtainment of rewards causing attenuation of the blink reflex. However, because of lacking appropriate neutral conditions, or of comparative analysis of startle magnitudes in neutral versus reward conditions, this claim is hard to evaluate. For example, in^10^ the difference between a neutral condition without any potential reward, and a condition with potential reward which did eventually not materialize was not statistically evaluated. Hence, previous studies have not clearly demonstrated whether motivational tendencies after reward feedback, as indexed by the startle reflex, are mainly characterized by appetitive responses to a gain or mainly by aversive consequences of reward omission, or by both. The present study addressed this issue using a design in which gain and reward omission can be compared separately to a neutral baseline, and in which reward expectation and actual reward obtained were orthogonally manipulated.

A wide variety of reward types and magnitudes are used across studies employing reward tasks. It is, however, unclear whether these different rewards have comparable subjective value on average. A second aim of the current study was to assess the subjective value of monetary rewards of varying magnitude. Startle-reflex modulation was used as a readout measure of the motivational state during the obtainment and loss of rewards of varying magnitude. Evidence from event-related potential (ERP), e.g.^12,13^ and imaging studies, e.g.^14,15^ suggests that at least some motivation-related processes operate in a graded (i.e., large reward > small reward > no reward) rather than all-or-none (i.e. any reward > no reward) fashion. However, reward magnitude was manipulated within-subject in these studies, leaving the possibility that valuation of rewards was scaled by the context of other rewards^16^. We, therefore, assessed motivational state during the obtainment and omission of rewards with varying magnitude in a between-subjects design.

A secondary issue in the present work concerns the phase in which participants anticipate a possible reward. A large body of studies investigating the anticipation of emotional pictures suggests that the startle response is predominantly potentiated during anticipation of an emotional event. Specifically, the startle reflex is potentiated during anticipation of both negative and positive picture slides compared to neutral picture slides^17–20^. This has been interpreted as indicating that startle reflexes during anticipation reflect global arousal, rather than motivational direction^20^. Startle facilitation has also been observed when participants anticipate a potential reward. During the anticipation phase of the Skolnick and Davidson^10^ lottery paradigm startle responses were potentiated when there was a chance to win money compared to when there was no chance to win money. So, in general, anticipating positive emotional information enhances startle magnitude, although startle *inhibition* has also been reported after cues signaling a potential reward compared to cues signaling a potential loss and neutral cues^21^. A secondary aim of the present study was therefore to replicate the anticipatory startle potentiation in a passive reward feedback-anticipation condition. Startle-reflex modulation was also evaluated during the presentation of an initial information screen indicating how much money could potentially be won during the current trial. This was done because there is a possibility that information about potential rewards in itself modulates appetitive-aversive motivation or arousal.

To address the above questions, a “head or tails game” was used, which was based on the task as implemented by Skolnick and Davidson^10^. Within this context a completely orthogonal design including the factors win potential (no money, money) and outcome (not won, won) was implemented. This design allows systematic investigation of the effect of money at stake and actual outcome on startle magnitude after feedback. Finally, we included a behavioral measure of appetitive motivation or arousal by analyzing the time needed to initiate the trial as a function of win potential and magnitude. During the head or tails game participants only had to press a button to spin a coin which could either land on heads or tails. This stands in contrast to paradigms in which participants may be led to believe that their actions influence reward probability. Examples are the lottery paradigm during which participants choose their ‘winning numbers’^10^, or a weather prediction task based on reinforcement learning^11^. Such paradigms may induce substantial variability in individual strategies driven by suspected associations between performance on the one hand and reward outcome on the other.

In sum, we set out to investigate startle modulation during anticipation and materialization (or not) of monetary rewards, varying reward magnitude between participants (Experiment 1). For reasons explained later, we attempted to replicate the major results in a within-subject design (Experiment 2).

## Experiment 1

### Methods

#### Participants

Fifty-seven volunteers were recruited using posters at the campus of Utrecht University. None of the participants used psychoactive medication and none of them had a history of psychiatric or neurological disorders. Participants were asked to refrain from consuming caffeine and smoking on the day of the experiment. Written informed consent was obtained and this study was approved by the medical ethical committee of the University Medical Centre Utrecht. The study was performed in accordance with the Declaration of Helsinki. All participants declared to have normal or corrected-to-normal vision. Participants were given a financial compensation of 6 Euro per hour and an additional monetary bonus dependent on the experimental condition they were assigned to. Participants were assigned to one of the following experimental conditions: 1 cent, 5 cent, 10 cent or 20 cent (reward magnitude; see Task an procedure). For the 1 cent, 5 cent, 10 cent and 20 condition the total amount of money “won” was fixed at € 0.28, € 1.40, € 2.80 and € 5.60, respectively. Reward magnitude groups were matched for gender, behavioral activation system (BAS) and behavioral inhibition system (BIS) scores and age. Table 1 displays the descriptive statistics for the four groups. None of the participants were aware of the aim of the experiment. One participant was excluded because of impaired hearing and 7 participants were excluded during analysis because they had too few valid startle responses in one or more conditions (see “Data processing”). Data of one participant were discarded because of a technical issue during the experiment. Therefore, the final sample consisted of 48 participants, 12 participants in each reward magnitude group.

**Table 1:** Descriptive statistics.

Group characteristics												
Study 1	GROUP										Study 2	
	1 cent		5 cent		10 cent		20 cent		F	*p*		
	Mean	SD	Mean	SD	Mean	SD	Mean	SD			Mean	SD
Males/females	12 (2/10)		12 (2/10)		12 (2/10)		12 (2/10)				24 (20/4)	
Age	22.71	2.48	21.59	1.85	22.26	3.30	22.76	3.50	0.43	0.73	22.6	4
BIS	20.50	4.64	21.83	3.95	20.75	3.72	21.58	3.37	0.32	0.81	20.88	3.37
BAS	39.75	3.55	39.83	5.83	39.08	4.54	39.33	4.89	0.07	0.98	40.04	3.58

#### Stimuli and psychophysiological recordings

The task was presented on a 16 inch Dell CRT monitor. Presentation of task and startle probes was controlled by Presentation® software (version 16.0, www.neurobs.com). Startle probes consisted of 50 ms, 102 dB white noise bursts presented binaurally through headphones (Sennheiser electronic GmbH, HD201, Wennebostel, Germany). During the anticipation phase of the task (see “Task and procedure”) a movie clip of a yellow spinning coin on a white background was presented (width: 4.4°, height: 3.2°).

The Active-Two system (BioSemi, Amsterdam, The Netherlands) with matching Ag/Ag–Cl FLAT type electrodes with a diameter of 11 mm was used to record the EMG data. Recording electrodes were placed over the orbicularis oculi muscle below the right eye, according to the guidelines given by Blumenthal et al.^22^. Common mode and ground electrodes were placed on the forehead. The EMG signal was sampled at 2048 Hz and filtered online with a 400 Hz low pass filter (DC).

#### Task and procedure

Upon arrival at the laboratory, participants were informed about the experiment and consent was obtained. Participants were seated in a dimly lit room, one meter in front of a monitor on which the task was presented. Because this experiment was part of a larger study, participants first filled in four questionnaires (including BIS/BAS to assess behavioral inhibition and behavioral activation, respectively) and were subjected to a different task for about 20 min. The BIS/BAS questionnaires^23^; Dutch translation by^24^ were used to test for between-group differences in aversive and appetitive tendencies (behavioral inhibition and behavioral activation, respectively), as BIS and BAS potentially interact with startle modulation during a reward task (see^8^). The last eight participants filled out the questionnaires in a prior session. This enabled us to match the experimental groups for BIS/BAS scores of the participants. Next, headphones were applied and participants received instructions for the experiment. Participants were told that they were going to play a head or tails game. Written instructions (on-screen) were as follows: ‘In this game you can win money during some trials. The amount of money you can win will be indicated each time. When indicated on the screen, you can press the spacebar to spin a coin. In case the coin lands on “tails” you win the indicated amount of money and when the coin lands on “heads” you will not win this amount of money. During this experiment you will hear short, loud noises.’

Just before commencing the head or tails game participants received nine startle probes while they looked at a fixation cross on the screen (habituation phase). The inter-startle interval during the habituation phase ranged between 20 and 23 s. The purpose of this habituation phase was to reduce the initial large startle responses. There was a small break of approximately 2 min at one-third and two-third of the head or tails game. Both rest breaks were followed by two startle habituation trials spaced apart by 10–13 s before the head or tails game continued. These habituation probes were presented in order to reduce the relatively large startle responses after a period of rest.

**Figure 1:**
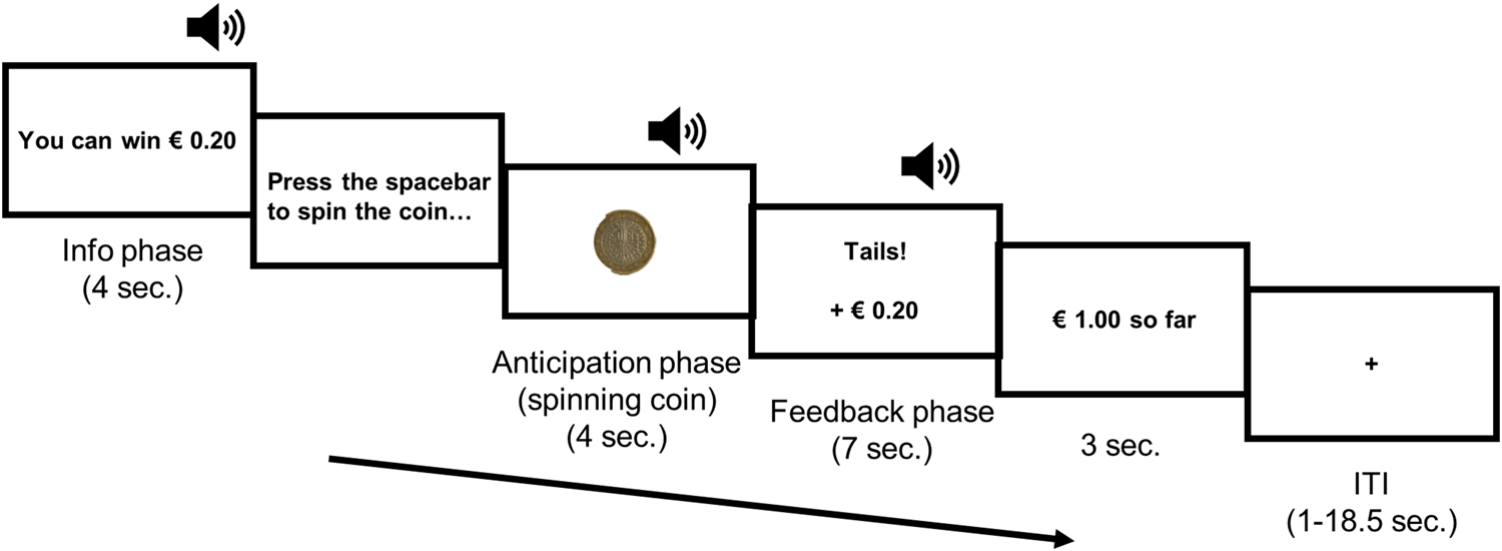
Overview of the task. The “head or tails paradigm” started off with an information screen indicating whether money could potentially be won or not (information phase). Next, participants pressed the spacebar to start a movie clip of a spinning coin (anticipation phase). This phase was followed by a feedback screen indicating whether the coin landed on heads or tails and how much money was won accordingly (feedback phase). Finally, an overview was given of the money won so far. Trials were separated by an intertrial interval (ITI) ranging between 1 and 18.5 s, to allow spacing of subsequent startle probes within the range of 25.25–33.25 s. Startle probes were delivered either during the information- (24 probes), anticipation- (24 probes) or feedback phase (48 probes; 12 per win potential and outcome condition). Note that unlike during the information- and anticipation phase, participants do know both win potential and trial outcome during the feedback phase. Therefore, the number of conditions/startle probes in each phase (before feedback (information and anticipation phase) and after feedback (feedback phase) is unequal. The amount of money at stake was either 0 Euro or a fixed amount of 1 cent, 5 cent, 10 cent or 20 cent, and was dependent on the reward magnitude group a participant was assigned to. The coin in this figure is only for illustrative purposes and not the actual coin shown to participants.

Figure 1 presents an overview of the task. A trial consisted of different steps to assess responses to anticipation of reward and response to reward feedback. The trial started with a screen presented for 4 s showing whether participants could win money or not that trial (information phase). The amount of money that could be won during a trial was either € 0,- or a fixed amount of either 1 cent, 5 cent, 10 cent or 20 cent, depending on the reward magnitude group a participant was assigned to. Next, a picture of a coin was presented together with an instruction to press the spacebar in order to spin the coin. This screen was present until the spacebar was pressed. Upon pressing the spacebar, a movie clip of a spinning coin was presented for 4 s (anticipation phase). The anticipation phase was followed by a 7 s feedback screen indicating whether the coin landed on heads or tails and how much money was won accordingly (feedback phase). The feedback phase was followed by a screen displaying the amount of money won so far for 3 s.

The task consisted of 112 trials of which 96 with a startle probe. On a given trial, startle probes were either presented during the information phase (24 trials in total; probes were delivered between 3.5 and 4 s after the start of the trial), or during the anticipation phase (24 trials in total; probes were delivered between 2.5 and 3 s after the coin began to spin), or during the feedback phase [48 trials in total, probes were delivered between 5 and 7 s following feedback; 24 probes were delivered after a positive outcome (‘Tails!’) and 24 after a negative outcome (‘Heads!)] of a trial in a counterbalanced fashion. The 16 trials without a startle probe were inserted to increase unpredictability of startle probe delivery. These trials were not considered in any of the analyses. Money could potentially be won in half of the trials, and the outcome was “tails” in half of the trials. Outcome in terms of heads (not won) and tails (won) was evenly distributed between trials in which money and no money was at stake.

Inter-trial intervals (ITI) consisted of a white fixation cross presented on a black screen. ITIs were adapted from trial -to -trial and ranged between 1 and 18.5 s in such a way that inter-startle intervals were kept within the range between 25.25 and 33.25 s, and were on average similar across conditions (29.25 s).

Participants were subjected to one of two task versions which differed with respect to the pseudo-random sequence of conditions. The conditions were evenly distributed during the experiment to avoid a differential effect of habituation on startle magnitudes for the different conditions. To summarize the number of trials and conditions: conditions were based on win potential (money [56 trials in total] or no money [56 trials in total] at stake), actual outcome (heads [56 trials in total: 28 no money and 28 money trials] or tails [56 trials in total: 28 no money and 28 money trials]) and phase (24 probes during the information phase, 24 probes during the anticipation phase, 48 probes during the feedback phase and 16 non-probed trials).

#### Data processing

Offline the EMG data were processed using Brain Vision Analyzer software (Brain products, Gilching, Germany), according to the published guidelines^22^ and studies previously published by our group, e.g.^25^. Trials were segmented, filtered with a 28 Hz high pass (24 dB/oct) and 500 Hz low pass (24 dB/oct) filter, rectified, smoothed with an additional 14 Hz (24 dB/oct) low pass filter and baseline-corrected (baseline from 30 ms before startle probe onset until 20 ms after startle probe onset). Peak magnitudes were scored within an interval between 25–120 ms after startle probe onset. Trials with a startle peak latency outside this 25–120 ms window or with EMG activity in the baseline period 2 SD greater than for the other trials in that subject were automatically detected and removed from the data. Trials in which there was less than 55% increase in activity within the 25–120 ms window with respect to the 50 ms peri-startle probe baseline period were marked as being a null response^25^, but not set to zero (i.e., the actual values were included in the analyses). Data of seven participants were discarded during the analyses, because more than half of the startle responses were invalid (null response or artifact) in one or more conditions.

Startle magnitudes were subsequently transformed to individual *z*-scores in order to remove variance due to large between-subject differences in overall startle magnitude. Consistent with previously published procedures^25,26^, *z*-scores were based on all individual habituation and experimental startle magnitudes recorded during the experiment. All statistical analyses were based on *z*-transformed data. Z-scores were transformed to *t*-scores for illustrational purposes (*z* * 10 + 50).

#### Statistical analysis

To explore the effect of reward magnitude and win potential on the time taken to initiate a trial, mean reaction times measured from the onset of the screen indicating that the spacebar can be pressed to the onset of the spacebar press were entered into a mixed-model analysis of variance (ANOVA). This ANOVA (SPSS version 20) included win potential (no money, money) as within-subject factor and reward magnitude (1 cent, 5 cent, 10 cent, 20 cent) as between-subjects factor.

Regarding EMG, for each participant *z*-scores were averaged across trials for each condition and phase. These values were entered in separate mixed-model ANOVAs (GLM, SPSS version 20) for the trials in which startle probes were delivered before receiving feedback (information phase and anticipation phase) and trials in which the startle probes were delivered after receiving feedback (feedback phase). The ANOVA for the feedback phase (main analysis) included win potential (no money, money) and outcome (not won, won) as within-subject factors and reward magnitude (1 cent, 5 cent, 10 cent, 20 cent) as between-subjects factors. The ANOVA for the pre-feedback phases (secondary analysis) included phase (information phase, anticipation phase) and win potential (no money, money) as within-subject factors and reward magnitude (1 cent, 5 cent, 10 cent, 20 cent) as between-subjects factors. The factor task version was initially included in these tests, but, as expected, no main or interaction effects with respect to task version were found for any of the ANOVAs. Therefore, data were collapsed across task versions.

A correlational analysis was performed to investigate whether a stronger reward-related effect on startle magnitude during the anticipation phase is associated with faster reaction times for initiating potentially rewarded compared to not rewarded trials.

Table S1 in the supplementary materials shows raw startle magnitudes in microvolts for each condition and each reward magnitude group separately. *Z*-scores were computed from all individual raw startle values including habituation trials (see “Data processing”). The across-subject averages for the means and standard deviations upon which *z*-scores were based amounted to 51.37 µV (SD = 40.35) and 32.41 µV (SD = 16.87), respectively.

Additional analyses were conducted using standardized startle magnitudes for which habituation trials were excluded from the *z*-transformation. The results of these analyses largely replicated the reported findings.

Alpha was set to 0.05. Post-hoc tests were corrected for multiple testing using Holm’s method^27^. A Greenhouse–Geisser correction of the *df*s was applied in case the assumption of sphericity was violated.

### Results

#### Behavioral data

A win potential (no money, money) by reward magnitude (1 cent, 5 cent, 10 cent, 20 cent) mixed-model ANOVA revealed that the amount of time it took participants to press the spacebar was significantly affected by win potential, *F*(1, 44) = 18.19, *p* < 0.001, η_p_^2^ = 0.293. Participants were significantly faster on trials during which money could be won (RT = 1068 ms, SD = 374 ms) compared to trials during which no money could be won (RT = 1200 ms, SD = 434 ms). There was no significant main or interaction effect of reward magnitude, *F*(3, 44) = 0.25, *p* = 0.859, η_p_^2^ = 0.017, and *F*(3, 44) = 0.72, *p* = 0.545, η_p_^2^ = 0.047, respectively.

#### Startle reflex data: information and anticipation phase

**Figure 2:**
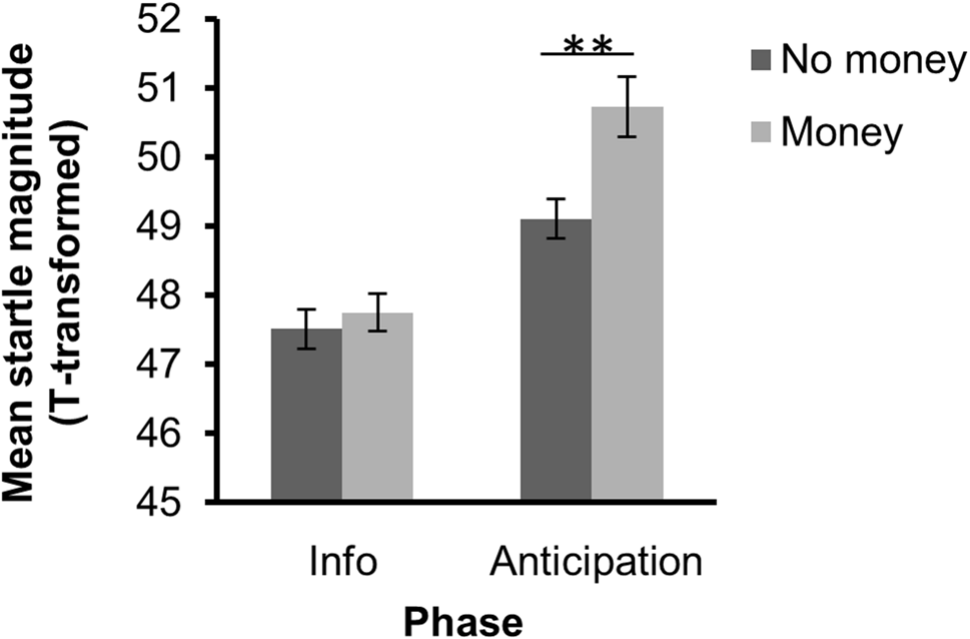
Mean startle magnitudes during the information phase and anticipation phase. Dark grey bars represent startle magnitudes in trials where no money could be won. Light grey bars represent startle magnitudes when there was a potential to win money. Startle magnitude was significantly increased when money could be won, compared to when no money could be won during the anticipation phase. The interaction between phase and win potential was significant (*p* = .045). Error bars represent ± standard error of the mean. **p < .01. Note that *z*-scores are transformed to t-scores for illustrative purposes.

A phase (information phase, anticipation phase) × win potential (no money, money) by reward magnitude (1 cent, 5 cent, 10 cent, 20 cent) ANOVA revealed significant main effects of phase, *F*(1, 44) = 34.44, *p* < 0.001, η_p_^2^ = 0.439 and win potential, *F*(1, 44) = 10.05, *p* = 0.003, η_p_^2^ = 0.186 (see Fig. 2, note that for illustrative purposes startle magnitudes are displayed as *t*-scores). Moreover, there was a significant interaction between phase (information phase, anticipation phase) and win potential, *F*(1, 44) = 4.27, *p* = 0.045, η_p_^2^ = 0.088. Separate *t*-tests for the information and anticipation phase comparing startle magnitude when money could be won and could not be won revealed only a significant difference between win potential conditions during the anticipation phase, *t*(47) = − 3.06, *p* = 0.004, but not during the information phase, *t*(47) = − 0.64, *p* = 0.524. No significant interactions with the between-subjects factor reward magnitude were found, *F*(3, 44) = 2.64, *p* = 0.061, η_p_^2^ = 0.153 (win potential × reward magnitude), *F*(3, 44) = 1.29, *p* = 0.289, η_p_^2^ = 0.081 (phase × reward magnitude), *F*(3, 44) = 1.06, *p* = 0.376, η_p_^2^ = 0.067 (phase × win potential × reward magnitude). There was also no correlation between the effect of win potential on startle magnitude during the anticipation phase and the effect of win potential on time to initiate the trial, *r*(46) = 0.172, *p* = 0.244.

Additional analyses for Experiment 1 with standardized scores omitting habituation trials show that the results only differ (not crucially) for the analysis of the pre-feedback probes. The phase x win potential interaction for the pre-feedback probes was now marginally significant, *F*(1, 44) = 3.67, *p* = 0.062, η_p_^2^ = 0.077. The significant main effects of win potential and phase were still present, *F*(1, 44) = 9.18, *p* = 0.004, η_p_^2^ = 0.173 and *F*(1, 44) = 46.96, *p* < 0.001, η_p_^2^ = 0.516, respectively.

#### Startle reflex data: feedback phase

**Figure 3:**
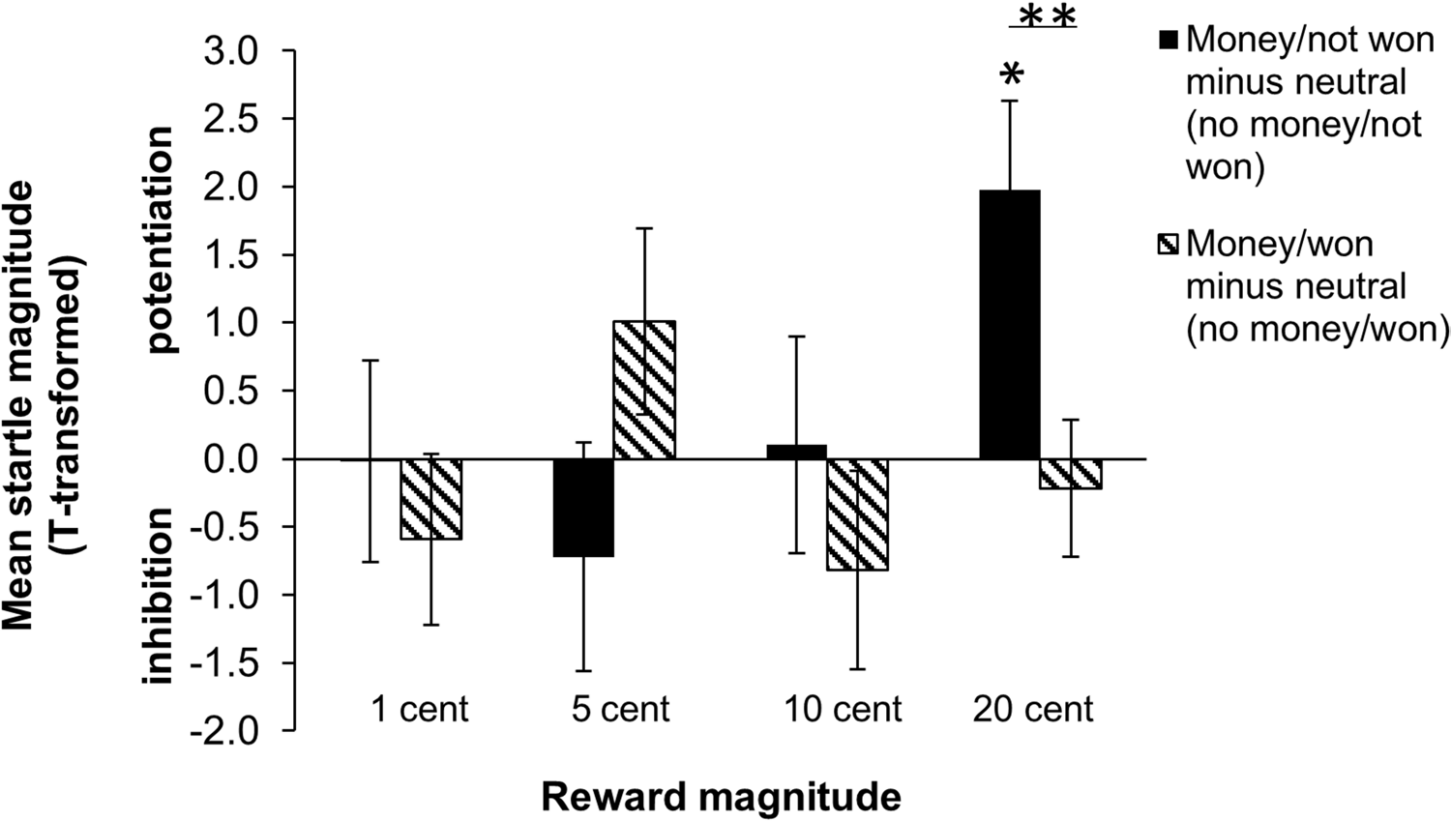
Bar graph showing the interaction between win potential and outcome on startle magnitudes for each reward magnitude group in the feedback phase of the task. Black bars represent the money potential/not won condition minus the respective neutral condition without potential gain (i.e., no money potential/not won). Striped bars represent the money potential/ won condition minus the respective neutral condition without potential gain (i.e., no money potential/ won). Specifically for the 20-cents condition startle magnitudes were significantly potentiated when money was at stake but the outcome was ‘not won’ relative to the neutral condition without potential gain. In contrast, startle was not inhibited when the outcome was ‘won’ relative to the neutral condition. Error bars represent ± standard error of the mean. Note that *z*-scores are transformed to *t*-scores for illustrative purposes. *p < .05, **p < .01. Figure S1 in the Supplementary materials presents the t-values for all conditions including the neutral conditions separately.

In the feedback phase, there was a significant three-way interaction between win potential (no money, money), outcome (not won, won) and reward magnitude (1 cent, 5 cent, 10 cent, 20 cent), *F*(3, 44) = 3.0, *p* = 0.041, η_p_^2^ = 0.169. Interactions between win potential and outcome for all reward magnitude groups are presented in Fig. 3. Note that for illustrative purposes startle magnitudes are displayed as *t*-scores.

Post-hoc analyses for all reward groups separately revealed a significant win potential x outcome interaction for the 20-cents group only, *F*(1, 11) = 10.05, *p* = 0.009, η_p_^2^ = 0.477. The win potential x outcome interactions for the other reward magnitude groups were not significant [*F*(1, 11) = 0.32, *p* = 0.583, η_p_^2^ = 0.028 (1-cents group), *F*(1, 11) = 3.04, *p* = 0.109, η_p_^2^ = 0.216 (5-cents group), *F*(1, 11) = 0.781, *p* = 0.396, η_p_^2^ = 0.066 (10-cents group)]. Breaking down the win potential × outcome interaction in the 20-cents group, in the trials in which money could be won, startle magnitude was marginally significantly larger when the actual outcome was ‘not won’ than when the outcome was ‘won’, *t*(11) = 2.46, *p* = 0.031 (Holms-corrected threshold for significance: 0.017). In contrast, when no money could be won, there was no significant difference in startle eye blink reflex between both outcomes, *t*(11) =  − 1.16, *p* = 0.271. Moreover, when comparing the difference in startle-reflex magnitude between the money and no money potential condition for each outcome, there was a significant difference in startle magnitude only when the outcome was negative (not won), *t*(11) = − 3.01, *p* = 0.012 and there was no significant difference when the trial was won, *t*(11) = 0.43, *p* = 0.674.

Note that the bar graphs in Fig. 3 suggest that the three-way interaction (win potential × outcome × reward magnitude) of the feedback startles may be driven by the 5-cents group showing a completely opposite pattern compared to the other reward magnitude groups. An exploratory win potential x outcome x reward magnitude ANOVA showed that the three-way interaction was no longer significant when the 5-cents group was excluded from the test, *F*(2, 33) = 0.84, *p* = 0.440, η_p_^2^ = 0.048.

### Discussion experiment 1

The main goal of the first experiment was to investigate appetitive and aversive motivation after feedback about whether a potential to win money had indeed materialized, relative to a baseline without potential gain. Startle blinks during the feedback phase were significantly modulated by feedback about whether the coin landed on heads (not won) or tails (won), as expected and observed in prior studies^10,11^, but only so when reward magnitude was at the highest level that was currently tested (20 cents per trial). This was manifest in the significantly larger startle magnitudes when there was a potential to win 20 cents but participants did not actually win the trial compared to a neutral baseline without potential gain.

In contrast to prior studies^10,11^, the present study used an orthogonal design including neutral trials in which participants knew they would not win money anyway (no money/won and no money/not won). This allowed us to explicitly test the difference between the money/not won condition versus the no money/not won condition, and the difference between the money/won versus the no money/won condition. We found that startles, and hence withdrawal motivation, were enhanced when 20-cents was anticipated but not won relative to when not anticipated and not won (i.e., 0-cents not obtained). In contrast, there was not at all significant startle inhibition when 20-cents was anticipated and won relative to the neutral baseline without potential gain (i.e., 0-cents obtained). The 20-cents/ not won condition clearly stands out from the other three win potential x outcome conditions, even though the difference between 20-cents/ not won and 20-cents/ won was not significant according to our strict Holms-dictated criteria (*p* < 0.031). Therefore, the findings support the idea that particularly for the 20-cents condition startle-reflex modulation after feedback is better characterized in terms of potentiation when a potential reward does not materialize, rather than in terms of inhibition as a result of the positive state.

Potentiation of the startle reflex after omission of a possible reward may have resulted from a primary defensive reaction in response to negative feedback, which is in keeping with the motivational priming theory^7^. It is furthermore possible that the startle reflex after reward omission was additionally potentiated as a result of an opponent process^28^ triggered by information displayed on the screen indicating that reward was at stake.

Kahneman and Tversky’s prospect theory^29^ states that the pain of a loss is experienced more intensely than the pleasure of a gain. To the extent that missing out on a potential appealing reward can be considered as experiencing a loss, this may provide an explanation for the observation that startle is potentiated after reward omission but not inhibited after winning. In addition, the stronger modulation in the 20-cents relative to the other reward-magnitude conditions, indicates that small rewards typically used in reward experiments may not be subjectively equivalent in value, which is relevant to future studies using monetary reinforcement. More specifically, a 20-cents reward may have higher subjective value on average than the lower-magnitude rewards, and hence, the “pain” of missing out on a 20-cents reward may be experienced more intensely than that of a lower-magnitude reward. This may also apply to other and larger rewards.

Figure 3 shows that the sign of the reward potential x outcome interaction was completely opposite for the 5-cents condition compared to the other conditions. The opposite pattern in the 5-cents condition may reflect a sampling error or the effect of some unknown characteristic of this condition. It is plausible that the interaction between reward magnitude, win potential, and actual outcome of the trial was driven by the opposite pattern in the 5-cents group rather than by the 20-cents group. This was suggested by the results of an explorative test in which the 5-cents group was not included. A follow-up study with both the 5 and 20-cents group was therefore warranted in order to confirm this hypothesis and to investigate if this interaction pattern for the 5-cents condition would replicate or whether it may have been due to a sampling error.

A secondary aim was to replicate the results of prior studies showing potentiation of the startle reflex during reward anticipation. As expected, startle-reflex magnitudes in the anticipation phase were enhanced when a reward was at stake compared to when no reward was at stake. The observation that the startle reflex was enhanced when participants anticipated a reward compared to the anticipation of no reward is consistent with a large body of literature showing that the anticipation of both appetitive and aversive pictures^17–20^, as well as potential rewards^10^ enhances the startle reflex. These results and the results of the current study are in keeping with the assumption that the startle reflex during anticipation of an emotional event mainly (but not exclusively^19^) reflects anticipatory arousal^20^. It is not likely that the observed effects during anticipation can be ascribed to differences in attention paid to the spinning coin movie clip. Effects of attention on startle magnitude have been found in an emotional picture-viewing study^30^, but these effects were most pronounced when probes were presented very early in the viewing interval (i.e., within 1 s after picture onset). In the current study anticipatory probes were presented between 2.5 and 3 s after movie clip onset. Furthermore, if more attention was allocated to the movie clip during potential reward trials this would likely have led to relative inhibition rather than potentiation of the startle reflex, as less resources would have been available for processing of the startle probe.

Participants were also faster to initiate the trial as soon as they knew that money was at stake compared to when they knew that nothing could be won. This effect of win potential on reaction times was, however, not associated with the effect of win potential on startle magnitudes.

Startle response magnitudes did not differ between the money and no money condition during the information phase, when participants just saw whether they could win money or not. This result may fit with an observation by Sege et al.^31^ who showed that startle potentiation during anticipation of positive slides was only present late in the anticipation phase*.* Alternatively, the video of the spinning coin with a predictable duration and anticipation of feedback regarding the reward that comes right after the end of the video may have increased physiological reactivity during the anticipation phase.

## Experiment 2

### Introduction

In Experiment 1 we aimed at comparing startle-reflex modulation between rewards of varying magnitude and found that this was specifically significant for the highest reward level (20 cents). The results of Experiment 1 support the idea that missing out on rewards with high absolute magnitude (i.e., at least 20 cents, the highest level currently tested) is experienced more intensely than missing out on rewards with lower absolute magnitude. However, it is possible that the 5-cents condition rather than the 20-cents condition drove the interaction between win potential × outcome and reward magnitude in Experiment 1. This hypothesis was tested by directly comparing the 5-cents condition with the 20-cents condition in a within-subject design.

Evidence suggests that reward value is scaled by the context of other reward options in an experiment^16^. The presence of a context of varying reward magnitudes may lead to more robust differences in valuation across the reward conditions. Therefore, in Experiment 2 a within-subject design was chosen to compare the higher (20 cents) with the lower (5 cents) reward magnitude. In the follow-up experiment with new participants we aimed to confirm the results of the analysis on reward feedback separately for the different reward magnitude conditions, and furthermore we aimed to re-investigate the pattern of startle-reflex modulation for the 5-cents condition.

In this follow-up experiment we used a very similar task in a larger number of participants. The within-subject design of Experiment 2 allowed us to investigate whether startle modulation is affected by relative reward magnitude. Based on the results of Experiment 1, we expected to find larger startle magnitudes after not winning a reward compared to neutral trials and trials during which a reward was won. We expected this effect to be stronger for the 20-cents compared to the 5-cents condition, based on Experiment 1 and because of the hypothesized scaling of reward value by the context of the other rewards. More specifically, the 5-cents reward was expected to have less subjective value, because even a larger reward could have been won or lost^12,14–16^. We also expected larger startle magnitudes during the anticipation of 5 and 20 cents compared to the anticipation of no reward, replicating our first experiment.

### Methods

#### Participants

Twenty-six new and naive participants were recruited via advertisement at the campus of Utrecht University and via advertisement on social media. The in/exclusion criteria were the same as in Experiment 1. Participants were asked to refrain from caffeine and smoking on the day of the experiment. Written informed consent was obtained and this study was approved by the local ethical committee of the Faculty of Social Sciences of Utrecht University. The study was performed in accordance with the Declaration of Helsinki. Participants received a financial compensation of 14.50 Euros or study credits. Participants additionally received the money won during the task (fixed at 5.25 Euros, which was unknown to the participants during the task). Two participants were excluded because more than half of the startle responses were invalid (null response or artifact) in one or more conditions. Therefore, the final sample consisted of 24 participants. Table 1 displays the descriptive statistics.

#### Stimuli and psychophysiological recordings

The startle stimuli and the psychophysiological recording procedures were the same as in Experiment 1.

#### Task and procedure

Subject eligible for participation signed the informed consent form and subsequently filled in the BIS/BAS questionnaire. The procedure was the same as in Experiment 1, unless stated otherwise. The head or tails paradigm was largely similar to the one used in Experiment 1. As in Experiment 1 a trial consisted of three phases: an information phase, anticipation phase (coin spinning), and a feedback phase. However, unlike in Experiment 1, startle probes were only presented during the anticipation and feedback phase, as startle modulation was only observed during these phases in Experiment 1. The task consisted of 126 trials, of which 108 with a startle probe. Thirty-six startle probes were delivered during the anticipation phase and 72 were delivered during the feedback phase, of which 36 following a positive outcome (tails) and 36 following a negative outcome (heads). Unlike in Experiment 1, reward magnitude was manipulated within-subject. The amount of money at stake (either 0, 5 cents, or 20 cents) as well as the outcome in terms of heads (not won) and tails (won) were counterbalanced across trials. Outcome was evenly distributed between trials in which 0 cents, 5 cents, or 20 cents was at stake. The timing of startle probe delivery, the duration of the events on the screen, and the average duration of the ITIs were the same as in Experiment 1. Three rest-breaks (self-paced) were provided after, respectively, 25, 50, and 75 percent of the trials.

To sum up the number of trials per condition, 36 startle probes were presented during the anticipation phase (12 probes were delivered while participants anticipated 0 cents, 12 while participants anticipated 5 cents, and 12 while participants anticipated 20 cents). Seventy-two probes were presented during the feedback phase (anticipate 0 cents/outcome not won [12 probes], anticipate 0 cents/outcome won [12 probes], anticipate 5 cents/outcome not won [12 probes], anticipate 5 cents/outcome won [12 probes], anticipate 20 cents/outcome not won [12 probes], anticipate 20 cents/outcome won [12 probes]).

#### Data processing and statistical analyses

The data processing steps in Brainvision analyzer were as described before. The raw startle magnitudes in microvolts were transformed to *z*-scores based on all individual experimental startle magnitudes excluding the habituation trials. These standardized values were averaged across trials for each condition and phase.

With respect to the EMG data, a repeated-measures analysis of variance (ANOVA) with the factor win potential (0, 5, 20 cents) was run for the anticipation phase. A win potential (0, 5, 20 cents) x outcome (not won, won) repeated-measures ANOVA was run for the feedback phase. Reaction time data were entered into a repeated-measures ANOVA with the within-subject factor win potential (0, 5, 20 cents).

A correlational analysis was performed to investigate whether a stronger reward-related effect on startle magnitude during the anticipation phase is associated with faster reaction times for initiating potentially rewarded compared to not rewarded trials.

Alpha was set to 0.05. Post-hoc tests were corrected for multiple testing using Holm’s method^27^. A Greenhouse–Geisser correction of the *df*s was applied in case the assumption of sphericity was violated.

### Results

#### Behavioral data

Reaction times were marginally significantly affected by the amount of money at stake, *F*(1.5, 34.8) = 3.26, *p* = 0.063, η_p_^2^ = 0.124 (Greenhouse–Geisser corrected). Reaction times tended to become shorter with increasing amounts of money at stake (M = 836, 798, and 784 ms, for the 0, 5, and 20-cents conditions, respectively).

#### Startle reflex data: anticipation phase

**Figure 4:**
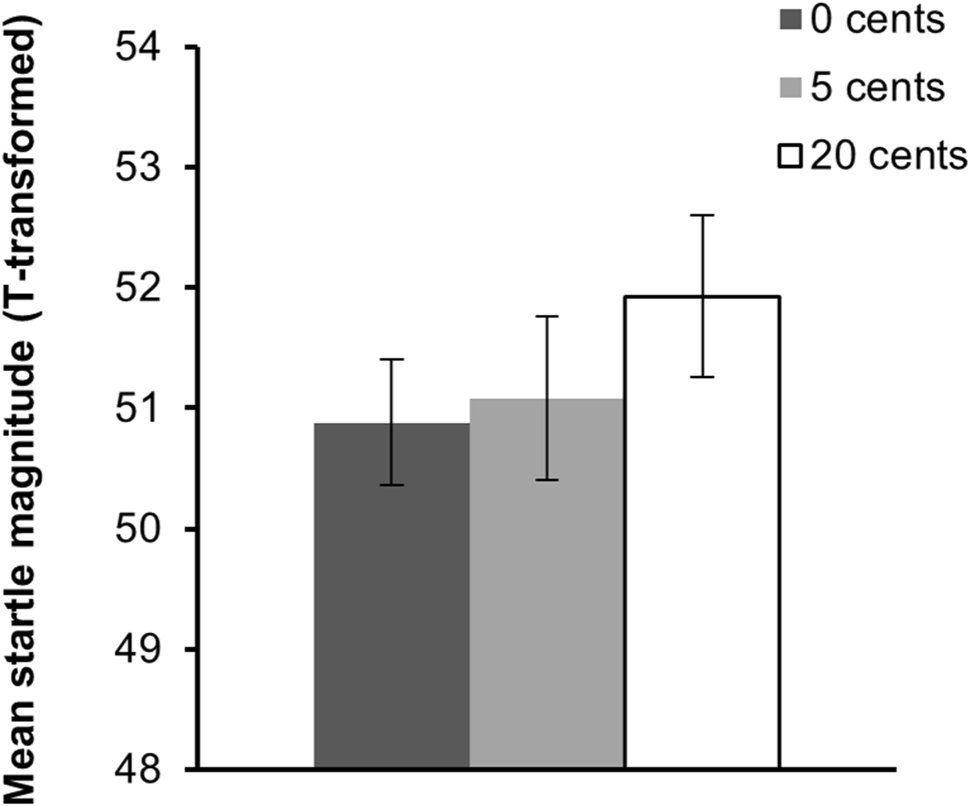
Mean startle magnitudes during the anticipation phase of the follow-up study. Startle magnitudes tended to be increased with increasing reward at stake. This effect was, however, not significant. Error bars represent ± standard error of the mean. Note that *z*-scores are transformed to *t*-scores for illustrative purposes.

Startle-reflex magnitudes during the anticipation phase were not significantly affected by the amount of money at stake, *F*(2, 46) = 1.20, *p* = 0.309, η_p_^2^ = 0.05. The pattern of results was, however, in the expected direction. That is, magnitudes tended to be larger with increasing amounts of reward at stake. See Fig. 4.

For none of the reward conditions significant correlations were observed between the effect of win potential on startle magnitude during the anticipation phase and the effect of win potential on time to initiate the trial, *r*(22) = 0.12, *p* = 0.586 (0 vs. 5 cents) and *r*(22) = 0.01, *p* = 0.949.

#### Startle reflex data: feedback phase

**Figure 5:**
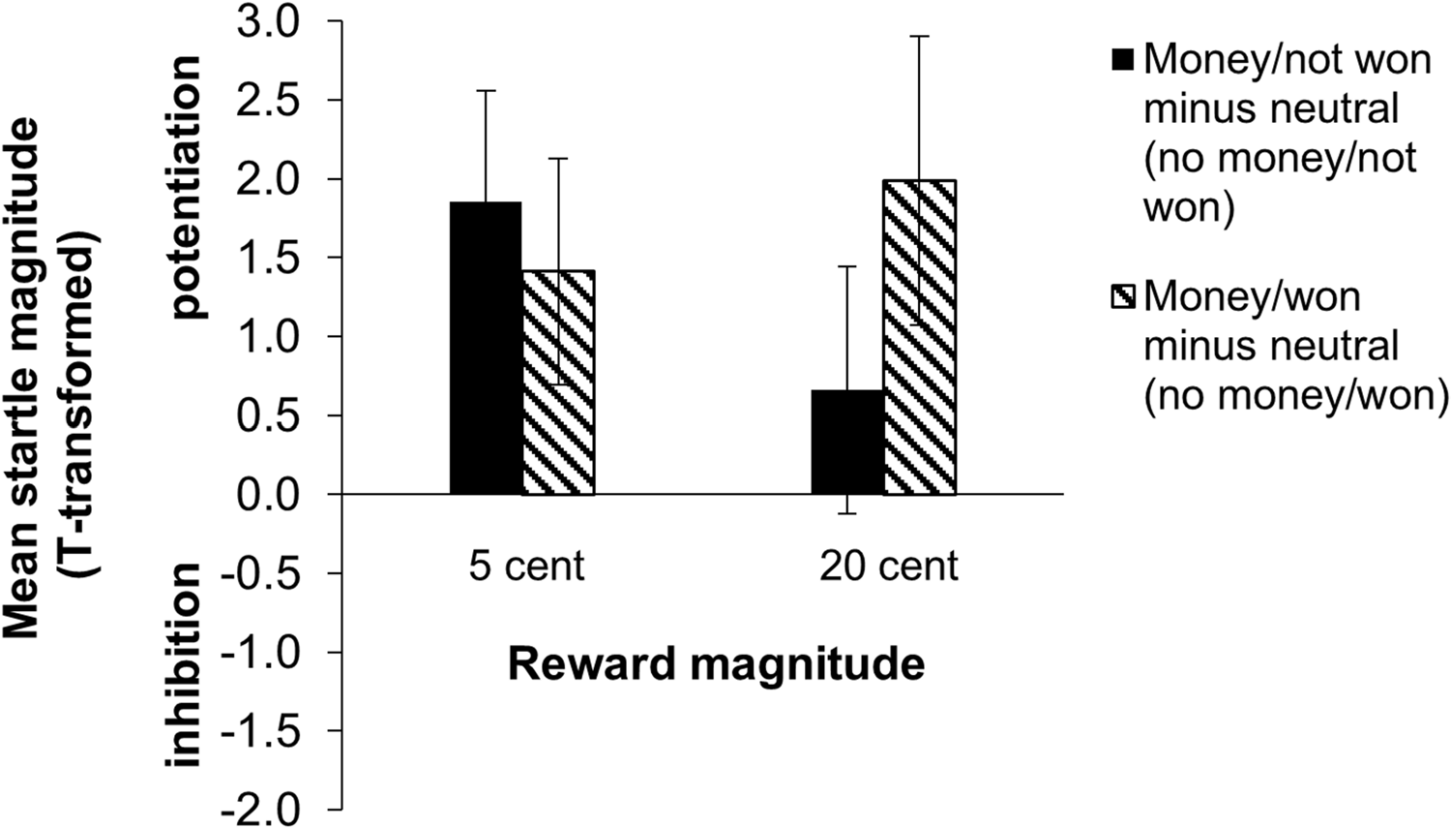
Mean startle magnitudes during the feedback phase of Experiment 2. Black bars represent the money potential/not won condition minus the respective neutral condition without potential gain (i.e., no money potential/not won). Striped bars represent the money potential/won condition minus the respective neutral condition without potential gain (i.e., no money potential/won). The win potential conditions (no money, 5 cent, and 20 cent) were manipulated within-subject. Startle magnitudes were significantly increased when 5 cents was at stake compared to the neutral condition without potential gain, irrespective of the actual outcome of the trial (in the Figure this can be seen as the average of the two bars on the left being significantly greater than 0). Error bars represent ± standard error of the mean. Note that *z*-scores are transformed to *t*-scores for illustrative purposes. Figure S2 in the Supplementary materials presents the t-values for all conditions including the neutral conditions separately.

The win potential x outcome ANOVA for startle magnitudes during the feedback phase revealed a significant main effect of win potential, *F*(2, 46) = 4.27, *p* = 0.02, η_p_^2^ = 0.156. See Fig. 5. Follow-up *t*-tests indicated that the startle magnitudes across both outcomes were larger for the 5 compared to 0-cents condition, *t*(23) =  − 3.20, *p* = 0.004, and marginally larger for the 20 compared to 0-cents condition, *t*(23) =  − 1.91, *p* = 0.068. There was no significant interaction with outcome, *F*(2, 46) = 1.80, *p* = 0.177, η_p_^2^ = 0.073.

### Discussion experiment 2

The main aim of this additional experiment was to replicate the pattern of startle modulation that we observed specifically for the 20-cents condition in Experiment 1. Specifically, we investigated whether startle modulation after outcome feedback is mainly driven by startle potentiation when a relatively large (compared to a smaller reward) reward does not materialize. The results for the feedback phase of Experiment 2 were, however, inconclusive with respect to this question.

Startle magnitudes during the feedback phase of Experiment 2 were larger for trials in which a 5-cents reward (and to a lesser degree a 20-cents reward) was at stake compared to trials without win potential, irrespective of trial outcome. This finding is puzzling and is clearly different from what we observed for the feedback phase in Experiment 1, in which the effect of win potential was dependent on the outcome of the trial. The pattern of startle modulation in the feedback phase of Experiment 2 seems more comparable to the pattern of startle modulation during the anticipation phase of Experiment 1. It is not clear why the findings of Experiment 2 are so different from those of Experiment 1. Though the task is very similar to the task we used in Experiment 1, a few differences may perhaps have contributed to these discrepancies. A first difference is that the rewards at stake were manipulated between subjects in Experiment 1, and manipulated within participants in Experiment 2. In Experiment 1 the only reward options that an individual subject encountered were 0 cents or a reward of fixed magnitude, whereas in Experiment 2 the trial types were more diverse (0 cents, 5 cents or 20 cents). Anecdotally, participants reported after the experiment that they did not always remember what the reward condition was by the time the feedback came. The inclusion of multiple reward conditions in Experiment 2 also led to differences in the reward potential: no reward potential ratio, which was 50:50 in Experiment 1 and 66:33 in Experiment 2. It may be speculated that the relatively high reward potential: no reward potential ratio in Experiment 2 caused less news-value for trials with reward potential, possibly leading to a loss of motivation or reduced attention to the reward information screens. This possibility is supported by the reaction time data showing that in Experiment 2 participants were only marginally faster on potential reward compared to no reward trials, and with the startle data during the anticipation phase indicating that startle magnitudes were not significantly increased when a reward was at stake. To the extent that startle during the anticipation phase reflects arousal, this suggests that the anticipation of reward did not lead to increased arousal relative to the anticipation of no reward.

The possible loss of motivation to obtain rewards, reduced attention to reward information, and the increased complexity of the task may explain why there was no significant interaction between reward potential and trial outcome for startle responses during the feedback phase in Experiment 2. These factors do, however, not explain why startle responses in the feedback phase were significantly affected by reward potential, irrespective of trial outcome, in a manner similar to the pattern observed for the anticipation phase of Experiment 1.

A second difference between the experiments was the moment of startle probe delivery. Startle probes in Experiment 2 were delivered either during the anticipation or the feedback phase, whereas in Experiment 1 they could also be delivered during the information phase. It is, however, unclear whether, and if so how, this difference between the two tasks contributes to the discrepancies between the results.

Finally, the results of Experiment 2 do not support our hypothesis based on the results of Experiment 1 that the difference in the startle-reflex magnitude between winning and not winning is driven by potentiation when a potential reward does not materialize, rather than by startle inhibition as a result of the positive state.

### General discussion

The main goal of this study was to investigate whether motivational tendencies after reward feedback, as indexed by the startle reflex, are mainly characterized by appetitive responses to a (potential) gain or mainly by aversive consequences of reward omission, or by both. Another main aim was to use the startle reflex as a readout measure of motivational state during the obtainment and loss of rewards with varying magnitudes in order to compare the average subjective value of these rewards. This was studied by comparing startle modulation during positive and negative outcome feedback relative to baseline conditions without potential gain, and by comparing startle-reflex modulation between different monetary reward conditions.

In Experiment 1 we found that specifically for the largest reward (20-cents) startle magnitudes were increased when there was a potential to win but participants did not actually win the trial compared to a neutral baseline without potential gain. In contrast, startle was not at all inhibited when money was at stake and the trial was won relative to a baseline without potential gain. Therefore, an interpretation in terms of potentiation in case of not obtaining a potential reward better fits the data pattern than one in terms of inhibition in case of obtaining (expected or not expected) reward. Additionally, as expected based on previous studies that show enhanced startle in conditions of emotional arousal, we observed startle potentiation during the anticipation phase when a reward was at stake.

However, neither of these results were replicated in our follow-up study. In Experiment 2, the main effect of reward potential that we expected during the anticipation phase was significant for the feedback phase. The non-replication of the startle potentiation for not won trials when a reward was at stake in Experiment 2 precludes drawing a conclusion regarding the main question about whether startle is mostly affected by the not won or by the won trials.

It almost seems that in the second experiment the feedback phase was the first time that participants actually noticed what was at stake. Why startle during feedback shows this effect of win potential irrespective of trial outcome rather than the previously published pattern (^10,11^, replicated in Experiment 1) is subject of speculation. Several changes in the experimental set-up (discussed above) cannot satisfactorily explain the discrepancies in results between the two experiments. Apparently, even though Experiment 2 was set-up as a replication of Experiment 1, these differences in the context in which the different conditions were experienced made the set-up less sensitive to reward effects at the feedback stage. The possibility that differences in motivation to obtain rewards contributed to the observed discrepancies between the results is supported by the reaction time data showing that participants in Experiment 1 were initially faster to spin the virtual coin as soon as they knew that money was at stake. This reaction time effect was, however, not significant in Experiment 2, suggesting that the appetitive tendency was relatively reduced in Experiment 2.

Some changes based on the apparent reduction in appetitive motivation in Experiment 2 can be recommended to increase participants’ motivation and task engagement. We recommend leaving the reward value of the current trial on-screen during the course of the trial. This prevents that participants forget the reward magnitude that is at stake. We also recommend emphasizing before the experiment that participants can take home the money that they win during the task. Furthermore, future studies may want to equalize the reward: no reward potential ratio, as this may have been a factor contributing to the discrepant results obtained in Experiment 1 and 2.

A limitation of the current study is that no participant ratings of motivation and arousal levels related to anticipating and obtaining/not obtaining a respective amount of money were collected. This makes it more difficult to make a causal interpretation of the reward-related startle modulation in these studies (i.e., are the effects driven by arousal or motivational value).

It should be noted that there is a possibility that the non-replication was due to the findings of Experiment 1 being chance effects rather than genuine effects. This alternative scenario is however less likely, because the pattern of results obtained in Experiment 1 (i.e., increased startle responses when anticipating a reward and increased startle responses for not winning compared to winning) was also observed in other studies^10,11^.

In conclusion, the finding that the startle reflex is potentiated during the anticipation of a reward compared to no reward seems relatively robust. In Experiment 1 startle magnitudes were potentiated when a relatively large reward (20 cents) was at stake but not won, compared to a neutral baseline without potential gain. The failure to replicate these effects in Experiment 2 may have been due to differences in motivation or task engagement. It could therefore be useful to repeat Experiment 2 with the proposed changes to the setup and procedure aimed at tackling the potential issue of reduced motivation. Perhaps this new experiment sheds more light on the question whether startle-reflex modulation after feedback is indeed characterized by the aversive consequences of reward omission for relatively large rewards. Together the data of the two experiments do not provide robust evidence for systematic increases in startle modulation with increased reward magnitude at stake. We therefore do not recommend using startle-reflex modulation as a means to assess the subjective value of rewards.

## Supplementary Information

Supplementary Information.

## Data Availability

Data are available upon request.
