# Supplementary material for: No consistent startle modulation by reward
Source: Sci Rep. 2021 Feb 23;11:4399. doi: 10.1038/s41598-021-82902-0 (PMC7902634; doi:10.1038/s41598-021-82902-0)
Supplement: Supplementary file 1 — Supplementary Information. [file 41598_2021_82902_MOESM1_ESM.pdf]

Supplementary materials of manuscript: "No consistent startle modulation by reward"

Iris Schutte, Johanna M.P. Baas, Ivo Heitland, & J. Leon Kenemans

Table S1

Mean raw startle magnitudes (and standard deviations) in microvolts for each condition and habituation phase (before task, after first break, after second break) for each reward magnitude group.

	**Reward magnitude group**			
	1 cent	5 cent	10 cent	20 cent
	Mean (SD)	Mean (SD)	Mean (SD)	Mean (SD)
**Condition**				
**Info phase**				
No win potential	28.00 (21.84)	55.30 (42.21)	49.54 (54.10)	38.94 (20.51)
Win potential	30.86 (20.47)	56.46 (43.03)	49.00 (51.88)	39.72 (26.66)
**Anticipation phase**				
No win potential	35.15 (24.28)	63.61 (52.85)	50.72 (49.66)	45.23 (25.58)
Win potential	38.92 (27.27)	76.66 (65.89)	52.30 (47.02)	45.50 (24.99)
**Feedback phase**				
No win potential - not won	30.07 (23.17)	64.69 (50.95)	47.74 (50.11)	37.92 (26.31)
No win potential - won	27.89 (18.41)	62.64 (49.62)	45.94 (43.22)	39.26 (26.14)
Win potential - not won	30.19 (20.49)	60.65 (45.10)	46.26 (41.69)	44.93 (29.80)
Win potential - won	27.84 (21.21)	64.74 (48.01)	45.19 (50.34)	39.39 (28.22)
**Habituation**				
Before the task (9 probes)	85.89 (47.58)	124.30 (73.30)	107.46 (80.46)	120.17 (64.03)
After first break (2 probes)	51.19 (42.27)	65.42 (60.09)	62.26 (69.82)	48.69 (36.47)
After second break (2 probes)	34.21 (31.74)	72.50 (64.07)	51.90 (50.32)	36.01 (19.65)

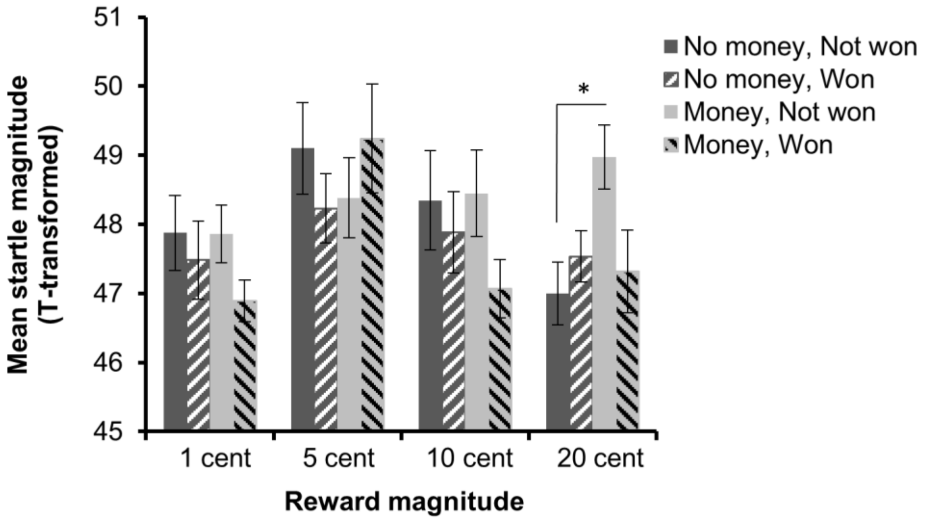

Figure S1. Bar graph showing the interaction between win potential and outcome on startle magnitudes for each reward magnitude group in the feedback phase of the task in Experiment 1. Dark grey bars represent no money potential and light grey bars represent potential to win money. Striped bars represent the outcome "won". Specifically for the 20-cents conditions startle was significantly potentiated relative to the baseline condition without potential gain when the outcome was 'not won'. In contrast to Figure 3 in the main article this Figure presents T values for all conditions including the neutral conditions (no money/ not won, and no money/ won) separately. Error bars represent ± standard error of the mean. Note that z-scores are transformed to T-scores for illustrative purposes. * $p < .05$.

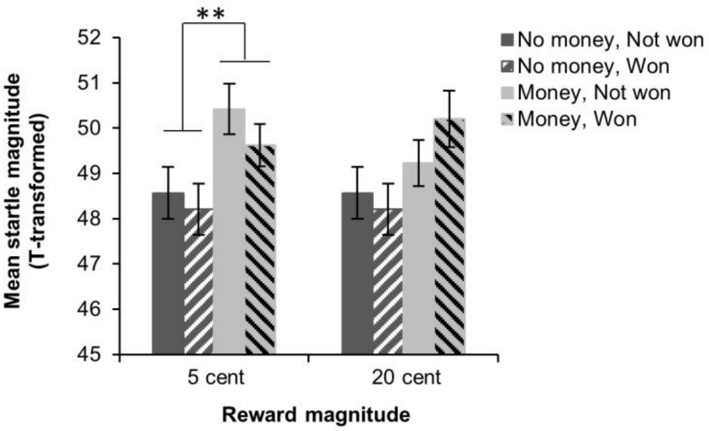

Figure S2. Mean startle magnitudes during the feedback phase of Experiment 2. Startle magnitudes during the feedback phase were differentially affected by the potential to win a reward, irrespective of positive/negative outcome (main effect of win potential, p = .02). This effect was driven by startle magnitudes across the won/ not won outcome being larger in the 5-cents compared to the 0-cents condition. The win potential conditions (0 cent, 5 cent, and 20 cent) were manipulated within-subjects. In contrast to Figure 5 in the main article this Figure presents T values for all conditions including the neutral conditions (no money/ not won, and no money/ won) separately. Error bars represent ± standard error of the mean. ** $p < .01$. Note that z-scores are transformed to T-scores for illustrative purposes. Note also that the outcomes in the 0-cents condition (no win potential) are in the Figure twice, visualized as “No money, Not won” and “No money, won”. This was done in order to make this Figure easier to compare with Figure S1.
